# Diet, Metabolism and Synaptic Function: Integrating Evidence Across Models in Neurodegeneration Research

**DOI:** 10.3390/biomedicines14051089

**Published:** 2026-05-12

**Authors:** Imogen L. Targett, John T. Hancock, Tim J. Craig

**Affiliations:** Centre for Biomedical Research, School of Applied Sciences, University of the West of England, Frenchay, Bristol BS16 1QY, UK

**Keywords:** high-fat diet, high-sugar diet, fatty acids, brain metabolism, neurodegeneration, diabetes, neurogenesis, neuroinflammation

## Abstract

The brain has a higher energy demand per unit weight than any other organ in the body; however, links between metabolism, diet and neurological function have historically been underexplored. This partly stems from early assumptions that brain metabolism is primarily dependent on glucose and ketone bodies, whereas more recent evidence indicates broader metabolic flexibility and complex cell-type specialisation. In the past few decades, brain metabolism has become increasingly recognised as relevant to neurological and mental health, and many neurodegenerative disorders are accompanied by changes in brain energy utilisation. In parallel, epidemiological studies associate hypercaloric dietary patterns and metabolic disorders—particularly type-2 diabetes mellitus—with increased risk of later cognitive decline and sporadic Alzheimer’s disease, although causal pathways remain difficult to establish in humans. In this narrative review, we summarise selected findings linking “unhealthy” diets to synaptic function, focusing on synaptic plasticity, neuroinflammation and adult hippocampal neurogenesis, and we distinguish between evidence from human observational studies and mechanistic insights from animal and cellular models. We also discuss candidate mechanisms—including insulin resistance-linked signalling changes, lipid-driven inflammatory amplification, oxidative stress, and altered lipid handling—that may contribute to synaptic vulnerability. Finally, we outline translational considerations and key knowledge gaps (including physiological exposure levels and heterogeneity of experimental paradigms) that currently limit inference from preclinical models to clinical intervention.

## 1. Introduction

The brain is the most energetic organ in the human body. At rest, it accounts for 20% of total energy consumption, despite comprising a mere 2% of body mass. This massive use of energy is hardly surprising: the 100 billion neurones in the brain have a vast membrane surface area and a highly dynamic membrane potential. Maintenance and control of this membrane potential by the regulated movement of ions, combined with the trafficking and recycling of untold billions of synaptic vesicles, places a high demand on ATP production. Fuel supply and metabolism in the brain are therefore of critical importance; however, this is a relatively poorly explored area with significant knowledge gaps.

The rise in public metabolic health issues such as type-2 diabetes mellitus (T2DM) has thrown brain metabolism into sharp relief; there is abundant evidence that metabolic disorders, including T2DM and metabolic syndrome, can lead to enhanced age-related cognitive decline; indeed, several studies have demonstrated an epidemiological link between these disorders and Alzheimer’s disease (AD), with T2DM sufferers 50–75% more likely to develop AD than the general population [1,2]. The reasons for this are currently unclear, but there is increasing evidence that fuel supply and metabolism alterations can lead to deficits in neuronal and synaptic function. In this review, we will examine this evidence and propose a model for how altered levels of sugars and fats in the brain can precipitate synaptic dysfunction. This is not intended to be a comprehensive review of all the work done in this area—such a view would be many hundreds of pages! Rather we are attempting to distil some key examples of the effects of diet on synaptic function, with an emphasis on more recent studies. In particular, we will focus on the cellular and animal studies which provide a more detailed mechanistic insight than observational studies in humans. We also acknowledge that several excellent reviews have already examined the effect of diet on cognition, e.g., [3,4].

Throughout this review, we use a simple working framework in which dietary excess can influence synaptic function through (i) relatively direct central nervous system (CNS) exposure and local cellular responses (e.g., altered substrate availability, lipid handling and inflammatory signalling within the brain), and/or (ii) indirect systemic metabolic mediation (e.g., peripheral insulin resistance, dyslipidaemia and chronic low-grade inflammation) that secondarily affects the CNS. These pathways are not mutually exclusive and likely interact (especially considering the timescale of human exposure). To avoid over-interpreting heterogeneous findings, we also aim to explicitly indicate the dominant evidence type being discussed—human observational, human interventional, animal in vivo, or cellular/ex vivo mechanistic—when drawing conclusions.

### 1.1. Neuronal Metabolic Pathways

The brain is dependent on glucose metabolism, although in certain circumstances (e.g., starvation) it can partially switch to ketones as a fuel source. This glucose dependence raises a paradox, as neurones themselves are only weakly glycolytic, due to constant degradation of the phosphofructokinase-fructose bisphosphatase-3 (PFKFB3) enzyme [5]. In recent years, a model has emerged whereby astrocytes metabolise glucose to lactate, which is then transferred to neurones and used as a substrate in the TCA cycle. This ‘lactate shuttle’ also links neuronal metabolism to neuronal activity [5]. Interestingly, a recent study overexpressed PFKBP3 in mice in order to increase neuronal glycolysis; these mice displayed cognitive decline and an inhibition of neuronal autophagy, suggesting that this weak glycolytic activity is required for normal neuronal functions [6].

The Jimenez-Blasco [6] study highlights the dependence of normal brain function on precisely calibrated metabolic pathways. It is therefore hardly a surprise that changes in neuronal metabolism are associated with a wide variety of diseases and neurological conditions. For a detailed review of brain metabolism and the roles of astrocytes, see Rose et al. 2020 [7].

An interesting question has always been whether neurones metabolise fatty acids as a fuel source. Traditional dogma states that this does not occur—however most of these papers are from many decades ago (e.g., [8,9]) and were often made on crude brain preparations. Indeed, several studies have shown that there is low or minimal expression of β-oxidation enzymes in mature neurones (e.g., [10]). However, a recent study [11] elegantly demonstrated that this is not the case, and that neurones can and do metabolise fatty acids derived from synaptic lipid droplets (LDs) in an activity-dependent manner. Again, this has relevance to disease, as changes in neuronal and glial cell lipid droplet presence and composition are observed in many neurodegenerative diseases [12].

Additionally, insulin signalling in the brain, long thought to be an ‘insulin-free organ’, has gained much attention recently. Not only is it important in synaptic plasticity [13], memory formation [14] and neurogenesis [15], but insulin resistance has been shown to occur in the brain in animal models of AD [16] and potentially in human patients [17], thus providing a potential reason for the link between T2DM and AD.

### 1.2. Linking Metabolism to Cognitive Decline

Many different epidemiological studies have linked high-fat (e.g., [18]), high-sugar (e.g., [19]) and high-processed-food diets (e.g., [20]) to increased dementia risk in humans. Most of these studies, however, rely on self-reporting of dietary intake, and out of necessity all are observational, which makes causality hard to prove. However, a more reliable epidemiological link has been shown between T2DM and AD specifically, with T2DM patients 50–75% more likely to develop late-onset AD. This makes T2DM the most significant non-genetic risk factor for AD.

Human studies are notoriously hard to control or show any kind of mechanistic or causal detail. For this reason, whilst we will refer back to human studies showing a link between metabolism and neuronal health, for the rest of this review we will focus on animal (mostly rodent) and cellular studies.

### 1.3. Synaptic Effects of High-Fat and High-Sugar Diets

As we will see in the following sections, hypercalorific diets (especially those containing high levels of fats) have profound effects on synaptic function, ranging from defective synaptic plasticity to structural alterations and inflammatory responses. Most of these studies have been carried out in highly controlled environments, e.g., rodent models or cellular systems, and it is uncertain how these observed effects translate to human physiology and pathophysiology. Nevertheless, the evidence summarised below indicates that synaptic function can be altered by dietary composition and metabolic context in experimental systems, and these effects are plausibly mediated—at least in part—by the dysregulation of intracellular signalling pathways that regulate synaptic plasticity and inflammatory responses.

## 2. Hypercalorific Diets

### 2.1. High-Sugar Diets

#### 2.1.1. Fructose

Several studies have demonstrated that diets enriched in specific sugars, especially sucrose and fructose, lead to reduced cognitive performance in rodents. Fructose is of particular interest due to its high levels in so-called Western diets, where it is often used as a cost-effective sweetener (being cheaper and sweeter than sucrose). However, several studies have linked increased fructose consumption to metabolic syndrome, fatty liver disease and T2DM [21,22,23].

For example, one study from 2015 demonstrated that a 7-day fructose-enriched diet resulted in decreased performance on a Morris Water Maze and that this was underpinned by reduced hippocampal synaptic plasticity [24]. Remarkably, this study showed a severe reduction in both hippocampal long-term potentiation (LTP) and long-term depression (LTD) in the fructose-fed rats, and a reduction in neurogenesis. These effects were accompanied by classical symptoms of metabolic syndrome, raising questions as to whether they are directly as a result of the effect of fructose on neuronal cells, or a secondary metabolic effect, e.g., insulin resistance. However, a later study intriguingly indicated that a high-fructose diet, especially when combined with chronic, unpredictable stress, increased calcium-calmodulin-dependent kinase II (CaMKII) phosphorylation and altered the expression of a variety of postsynaptic proteins, e.g., increased drebrin and decreased gephyrin [25]. Given the importance of these proteins in synaptic plasticity, this gives a possible mechanism for the results of Cisternas et al. [24]. Together, these studies are consistent with fructose-associated alterations in synaptic plasticity and synapse-related protein expression in rodents; however, interpretation depends strongly on whether effects are mediated indirectly via systemic metabolic disruption (e.g., insulin resistance/oxidative stress/inflammation) versus more direct CNS exposure, and this distinction should be treated cautiously unless exposure and metabolic endpoints are measured in parallel. Indeed, it seems more likely that fructose acts on neurones at least partially indirectly, for example, via the induction of oxidative stress [26] or the activation of neuroinflammatory pathways [27]. However, evidence of direct action does exist: a short-term (1 week) high-fructose diet in rats has been shown to increase neuronal expression of the fructose transporter, GLUT5 [28], facilitating fructose entry into neurones. Given the demonstrated induction of insulin resistance in other cell types [29,30,31] by direct fructose exposure, it seems likely that such a mechanism may also operate in neurones. As neuronal insulin signalling is increasingly regarded as critical for synaptic plasticity [32], this has obvious implications for neurodegenerative disease and cognitive decline. Interestingly, this study also showed a reduction in axonal and mitochondrial markers in the hippocampus of these animals, and these changes occurred without the induction of metabolic syndrome, therefore implying an acute, possibly direct neuronal effect.

It is important to emphasise that not all studies have shown detrimental effects of fructose. Indeed, one study even demonstrated a reduction in neuronal inflammation in response to a high-fructose diet [33]. Overall, many studies report adverse behavioural and synaptic outcomes under high-fructose paradigms, but discrepant findings likely reflect differences in exposure duration, age/sex, stress context, behavioural assays, and the extent to which systemic metabolic dysfunction is induced—highlighting the need to interpret “fructose effects” as context-dependent rather than uniform.

#### 2.1.2. Sucrose

Diets high in sucrose, the most commonly used sugar in foods, have also shown profound neurological effects in animal models. Unlike the studies using fructose, above, these studies tend to involve a longer-term, chronic exposure to elevated sucrose levels, possibly more representative of human diets. Typically, these dietary regimes involve around 6 months of exposure where sucrose is given ad libitum as a 10–20% solution.

Several of these studies have demonstrated a reduction in working memory, spatial reasoning and hippocampal long-term potentiation induced by such diets in rodents [34,35,36,37]. These hippocampal LTP effects were similar, although less pronounced, than those for fructose-exposed rodents [24], and seem to underlie the memory defects identified. It was also notable that most of these studies demonstrate increased body weight, reduced glucose tolerance and increased insulin resistance as a result of these diets, suggesting that at least some neuronal/synaptic effects may be mediated by broader metabolic dysregulation (e.g., impaired glucose tolerance and insulin resistance) rather than a direct effect of sucrose exposure on neurons per se, although the relative contribution of direct versus indirect mechanisms remains difficult to disentangle across paradigms. One study demonstrated increased excitability of the AgRP neurones of the hypothalamus, which are involved in appetite and body weight regulation [38]. Thus, high sugar consumption seems to ‘prime’ the brain for excess calorie intake as well as dysregulating metabolism in general. This suggests that a ‘vicious circle’ feedback loop of excessive calorie intake, obesity, hyperphagia and metabolic syndrome could be induced by short-term exposure to a hypercalorific diet. Another key observation from more than one study (e.g., [37]) is that the age of high-sucrose exposure is critical—with juveniles experiencing greater and longer-lasting deficits than adults. This obviously has implications for human health, given the prevalence and aggressive marketing of highly calorific products to young people.

It is important to note that not all studies have shown similar results—for example, one study from 2022 [39], where rats were fed on 10% sucrose for 5 weeks, showed no differences in any of the behavioural tests used, despite the rats showing a high calorie intake and increased body weight. However, it should be noted that the tests used here were different from the studies above, e.g., using touch screens for object discrimination rather than classical spatial memory such as the Morris Water Maze. It should also be noted that this study did not look at metabolic parameters—therefore, it is possible that the sucrose treatment regime did not induce the metabolic dysfunctions required to cause neuronal deficits.

### 2.2. High-Fat Diets

High-fat diets (HFDs) are perhaps a more common paradigm of ‘unhealthy’ diet than those high in sugars. A typical HFD for rodents consists of 60% of daily calorie intake from fat, although the composition of the fats used is often not specified. Additionally, there is considerable variation in the duration of such diets—ranging from just a few days to many months. Therefore, “high-fat diet” paradigms vary substantially across studies (fat source and saturation, carbohydrate content, caloric density, duration, and the age/sex/strain of animals), which can shift outcomes from primarily CNS-facing substrate effects to predominantly systemic metabolic dysfunction. Where possible, interpretation should consider whether obesity and insulin resistance are present, and whether endpoints reflect acute synaptic physiology, longer-term circuit remodelling, or behavioural performance.

Whilst the specific mechanistic studies we discuss here are all on rodent models, there is abundant evidence in humans of elevated levels of a variety of lipids in both AD [40] and Parkinson’s disease [41], supporting an association between altered lipid profiles and disease state in these conditions.

Several studies have demonstrated that synaptic dysfunction can be induced by short-term exposure of rodents to HFD. Changes observed include reduced hippocampal synaptic density and increased blood–brain barrier permeability [42], reduced hippocampal LTP and fEPSPs [43] and impaired memory formation [44]. Interestingly, some of these events were shown to be triggered by neuroinflammation, particularly in aged rats [43,45] (see below), whereas in other studies of adolescent rats, immature (DCX^+^) neurones in the hippocampus showed reduced dendritic complexity concurrent with lower BDNF levels [46]. This is particularly interesting, due to the potential effects of diet and metabolism on adult hippocampal neurogenesis (AHN), and the potential involvement of this in the early pathology of AD. This will be discussed in detail in subsequent sections. Importantly, a short-term HFD does not result in obesity or overt metabolic disorders, likely indicating direct effects of such a diet on the brain. Another interesting observation from several of these studies is that the effects of a short-term HFD are reversible.

Whilst some studies use short-term (a few days to 2 weeks) treatment as detailed above, the majority of studies using HFDs take place over many weeks. This is likely to be more representative of the long-term exposure seen in ‘unhealthy’ human diets and will generally include the effects of systemic metabolic dysfunction. As such, although these studies are arguably more realistic, the mechanistic reasons for their effects are more complex to unpick.

Similarly to short-term treatments, the vast majority of long-term HFD studies performed on rodents have shown pronounced neuronal effects. One particular study demonstrated that an 8-week HFD in mice resulted in decreased synaptic plasticity, dendritic spine density and autophagy in the hippocampus, possibly due to dysfunctional Wnt5a signalling [47]. These mice also exhibited depressive-like behaviour. Intriguingly, most of these deficits could be reversed upon regular exercise. Other studies have shown memory impairments due to altered activity of neuronal circuits [48], abnormal cortical plasticity and motor control [49] and alterations to reward/appetite circuitry in the hypothalamus, resulting in hyperphagia [50]. This latter observation is strikingly similar to that shown for sucrose in the previous subsection and perhaps represents a response to a hypercalorific diet rather than fat specifically. Specifically related to AD, more than one study has noted an exacerbation of disease pathology in AD-model mice when fed a high-fat diet [51,52].

From all these studies, it appears that an HFD has deleterious effects on the brain; however, the specific reasons and mechanisms for this are not clear purely from these studies. We will now turn our attention to the molecular mechanism that may underpin these diet-induced effects, focusing on the synaptic effects of fatty acids.

#### 2.2.1. Neuronal Effects of Elevated Free Fatty Acids

Free fatty acids (FFAs), the main constituent of triglyceride energy stores, are a major energy substrate for most areas of the body. The brain was long thought to be an exception to this, with neurones reported to be unable to metabolise fatty acids at any appreciable level. However FA β-oxidation accounts for approximately 20% of the adult brain’s total energy production, primarily carried out by astrocytes [53], and neurones possess some components required for FA β-oxidation including long chain acyl-CoA synthetase, an enzyme which initiates FA β-oxidation [9]. Moreover, a recent paper demonstrated that neurones use FFAs as a fuel source [11]. It is also clear that they readily cross the blood–brain barrier [54,55]. There are also local lipid trafficking mechanisms between astrocytes, microglia and neurons. Importantly, astrocytes appear to be better equipped for substantial β-oxidation and can buffer lipid load and redox stress, whereas neurons may rely more on tightly regulated lipid utilisation and activity-linked trafficking (e.g., lipid droplets/lipid transfer) and may therefore be more vulnerable to dysregulated exposure. Therefore, it seems likely that altered levels of FFAs will affect neurological function, either by direct effects on neurones themselves, or via alterations in neuronal–glial interactions. Explicitly distinguishing which cell type is being discussed (astrocyte, neuron, microglia, or endothelium) is essential when interpreting mechanisms involved in fatty acid-mediated neurological dysfunction.

Elevated FFAs can occur in a variety of conditions, including various lipidaemias, obesity and T2DM. These last two conditions are of most interest, as both are linked to cognitive impairment. As mentioned previously, T2DM confers a 50–75% greater risk of AD, and obese individuals are more likely to suffer from age-related cognitive decline even in the absence of overt dementia [56]. Individual FFAs are not typically measured in clinical studies; however, several studies have reported elevated levels of the two most abundant fatty acids, oleate (monounsaturated) and palmitate (saturated), in T2DM [57]. Estimates vary greatly but are generally in the range of 60–300 µM in healthy individuals, with oleate levels being lower than those of palmitate [58,59,60]. In T2DM, these levels are often increased by up to 50%; however, this is not always observed when oleate is examined. Therefore, many studies have investigated the effects of elevated palmitic acid levels on neurones (typically in the range of 100–250 µM) in attempts to explain the link between HFD, T2DM and cognitive decline.

It should be noted that it is extremely difficult to obtain reliable estimates of the levels of FFAs that neurones themselves are exposed to in situ. However, one study assaying the FFA content of the cerebrospinal fluid (CSF) of obese and T2DM patients with mild cognitive impairment (MCI) concluded that these conditions led to levels of palmitic acid of around 20 µM, compared to ~8 µM for healthy controls [61]. A recurring challenge, therefore, is the mismatch between palmitate concentrations used in vitro (often 100–250 µM) and the lower concentrations reported in human CSF (e.g., ~20 µM in the cited study). This discrepancy does not invalidate mechanistic findings, but it does mean that some observed synaptic effects may reflect supraphysiological exposure, acute stress responses, altered albumin binding in culture conditions, or experimental extremes that are not routinely achieved in vivo. Few studies have prioritised chronic, lower-dose paradigms; however, anchoring exposure to measured CNS bioavailability could strengthen translational inference in future studies.

#### 2.2.2. Palmitate-Induced Synaptic Dysfunction

Many different studies have linked increased palmitate levels to various degrees of neuronal dysfunction. Due to the sheer number of studies carried out in this field, we will not describe them all in detail here; however, several excellent reviews of this subject exist, e.g., [62,63]. There are several different mechanisms by which this has been proposed to operate.

##### Dysregulation of Palmitoylation

Whilst palmitic acid is primarily known as a dietary/serum FFA, it is also a key prenyl group for post-translational modification. Palmitoylation is abundant and critical for neuronal function—many pre- and postsynaptic proteins are palmitoylated either tonically or in response to cellular processes [64]. One such protein is the AMPAR subunit, GluA1, the palmitoylation of which is critical in regulating its surface trafficking during LTP [65]. At least two studies have demonstrated, using animal models, brain slices and isolated neurones, that exposure to increased palmitic acid (at around 200 µM for the isolated cells) disrupts GluA1 surface trafficking and therefore long-term potentiation [32,66]. In one of these studies [32], this was demonstrated to be due to excessive palmitoylation, and it seems reasonable to assume that it is the mechanism in the other study too. Excessive palmitoylation may drive a general dysregulation of synaptic and neuronal network plasticity, as a recent study has shown that this post-translational modification is very tightly regulated both spatially and temporally [67]. Therefore, it could be easily imagined how even subtle perturbations to this process due to enhanced substrate availability could have deleterious neuronal effects.

##### Insulin Resistance

Fatty acids have long been implicated in the induction of hepatic and skeletal muscle insulin resistance [57] and, as stated earlier, neuronal insulin resistance has been demonstrated in models of AD, suggesting a role in disease pathology. Treatment of primary neurones and neuronal cell models with high doses of fatty acids (generally ~200 µM for 24–48 h), including palmitate and oleate [68,69], induces insulin resistance via a variety of mechanisms, including metabolism-linked alterations in Ca^2+^ signalling and induction of neuroinflammatory pathways. This latter mechanism seems to involve the Toll-like receptor 4 (TLR4); although palmitate is not generally considered a direct TLR4 ligand, it may amplify or sensitise TLR4-associated inflammatory signalling indirectly, for example, by promoting pro-inflammatory co-factors (e.g., complexes involving fatty acid-binding proteins such as fetuin-A) [70] or by altering membrane organisation (including increased clustering of TLR4 into lipid rafts), thereby lowering the threshold for downstream NF-κB activation [71].

Another potential mechanism of palmitate-induced insulin resistance is ceramide synthesis. Ceramides are key intermediates in sphingolipid synthesis which, in turn, is required for myelin synthesis and therefore occurs at a high level in the brain. However, increases in ceramide synthesis, for which palmitate supply is a limiting factor, are strongly implicated in insulin resistance in hepatocytes and muscle cells [72]. This is due to the activation of protein phosphatase 2A (PP2A) by ceramide, which catalyses the dephosphorylation of several key kinases in the insulin signalling pathway, including Akt, resulting in insulin resistance. Ceramides have also been demonstrated to have a key role in the induction of insulin resistance in neurones by high-fat diets [73] and also in hyperinsulinaemia-induced mitochondrial dysfunction [74]. Importantly, alterations in ceramide production/accumulation have also been implicated in the pathology of Alzheimer’s disease in humans (e.g., [75,76]). Therefore, whilst the direct link between fatty acids, ceramide, insulin resistance and neurodegeneration has not been definitively made, the evidence very much points to this as a pathological mechanism. Given the recognised importance of insulin signalling in synaptic plasticity (which is inhibited in early AD), this may represent an early, even prodromal, stage in AD and could potentially be used as a biomarker.

##### Alzheimer’s Disease Markers

Several studies have also demonstrated a potential role for palmitic acid exposure in Aβ processing and tau phosphorylation, the two main candidates for pathological drivers of AD. A 2005 study [77] demonstrated that both palmitic and stearic (another SFA) acids can induce tau phosphorylation at AD-associated epitopes in rat primary neurones. This was also shown in a study in 2024 [78], whose data suggested that this increase in tau phosphorylation was due to an increase in GSK3β activity, a mechanism that fits well with insulin resistance, as GKS3β is phosphorylated (and therefore inactivated) by Akt. Similarly, other studies have shown that palmitic acid can increase Aβ production, potentially via upregulation of the β-secretase, BACE1 [79,80,81].

##### Neuroinflammation

Neuroinflammation is an extremely important topic for synaptic function; however, a detailed discussion of this process is beyond the scope of this review. Instead, we will focus on the evidence that palmitate can influence neuroinflammation in animal and cellular models.

Many different lines of evidence point to an inflammatory effect of palmitate on microglial cells and astrocytes. For example, studies exposing the microglial cell lines BV2 [82] and HMC3 [83] to differing levels of palmitate (80–200 µM) resulted in the induction of an inflammatory phenotype, measured by increased expression/secretion of cytokines, e.g., IL-1β, IL-6, and MCP-1, and increased signalling via NF-κB. Some of this effect was mediated by TLR4 (see subsection on insulin resistance). Similarly, a large meta-analysis of microglial activation via palmitate demonstrated consistent findings in the literature linking elevated palmitate levels to enhanced inflammatory cytokine signalling [84]. Studies on astrocytes have also yielded similar results, e.g., [85]. Given the critical joint role of astrocytes and microglia in mediated neuroinflammation, these studies together paint a picture whereby elevated palmitic acid strongly enhances neuroinflammation, at least in vitro.

In vivo studies have demonstrated a similar correlation between a high-fat diet, or palmitate itself, and neuroinflammation. One study demonstrated an increase in saturated fatty acids, including palmitate, in the hippocampus of aged mice fed a high-fat diet. These mice also showed an increase in pro-inflammatory markers, including IL1-β [86]. Additionally, intracerebrovascular infusion of palmitate in mice has been shown to increase inflammatory signalling in the hippocampus, measured by increased TNF-α signalling [61]. These mice also displayed reduced synaptic plasticity and memory defects. Many other studies have shown similar relationships between high-fat diets and neuroinflammation [87,88]. Intriguingly, these last two studies also show marked sex differences, with female mice far less susceptible to high-fat-diet-induced neuroinflammation than males.

This is a well-established observation in mice, the reasons for which are unclear. Across many diet-based paradigms, sex is an important moderator of CNS outcomes, including neuroinflammation, synaptic plasticity and adult hippocampal neurogenesis. In the HFD studies cited above, females can show reduced susceptibility to neuroinflammatory stress relative to males, potentially reflecting differences in immune function, adipose distribution, peripheral metabolic responses, and the immunomodulatory actions of ovarian hormones (e.g., oestrogens). However, sex effects are not uniform: protection observed in young adult females may diminish with ageing or following loss of ovarian hormones, and some outcomes (e.g., neurogenesis) can show opposite directionality depending on exposure window and functional readout. Importantly, many behavioural studies use single-sex cohorts, and many in vitro mechanistic studies use immortalised cell lines, and therefore cannot resolve sex-dependent mechanisms.

Taken together, these data support both correlational links (dietary fat ↔ inflammatory markers) and, in some cases, causal contributions of lipid exposure to inflammatory signalling and synaptic impairment. However, the strength of causal evidence varies by model (cell line vs. in vivo), exposure route, and whether systemic metabolic dysfunction is present. The exact mechanisms linking neuroinflammation and high-fat diet remain incompletely explored; nonetheless, given the prominent role that neuroinflammation plays in many neurodegenerative diseases, coupled with the established link between such diets and cognitive decline in humans, these pathways remain strong candidates for contributing to synaptic vulnerability.

##### An Integrated Model of Palmitate-Driven Synaptic Vulnerability

Integrating the findings summarised above (and in Table 1), we propose a working model whereby elevated palmitate—arising from dietary saturated fat, obesity/T2DM-associated dyslipidaemia, and/or local CNS lipid handling—can engage multiple, partially overlapping pathways that are each supported to different extents across experimental systems. This model is intended as a conceptual scaffold for organising heterogeneous findings rather than a single evidence-equivalent causal chain. First, increased palmitate availability may perturb *S*-palmitoylation dynamics of key synaptic proteins (e.g., GluA1), disrupting receptor trafficking and plasticity. Second, palmitate exposure can promote neuronal insulin-signalling impairment via lipid-driven stress pathways, including indirect amplification of TLR4-associated inflammatory signalling and ceramide accumulation (with downstream PP2A/Akt/GSK3β effects that also intersect with tau phosphorylation pathways discussed later). Third, mitochondrial stress and oxidative damage—whether driven by lipotoxicity in neurons or by altered astrocytic buffering capacity—can further reduce synaptic resilience. Finally, palmitate can act on glial cells (microglia and astrocytes) to amplify cytokine signalling (e.g., TNF-α/IL-1β/NF-κB), creating a feed-forward inflammatory cascade that impairs synaptic function and memory in vivo. The relative contribution of these pathways is likely to depend on cell type, exposure duration (acute high-dose vs. chronic low-dose), albumin binding/bioavailability, and whether systemic metabolic dysfunction is present. Accordingly, we interpret individual mechanistic studies as identifying plausible components of a multifaceted, rather than a linear, pathway, and we would like to emphasise that different paradigms may preferentially sample different nodes within it.

**Figure 1:**
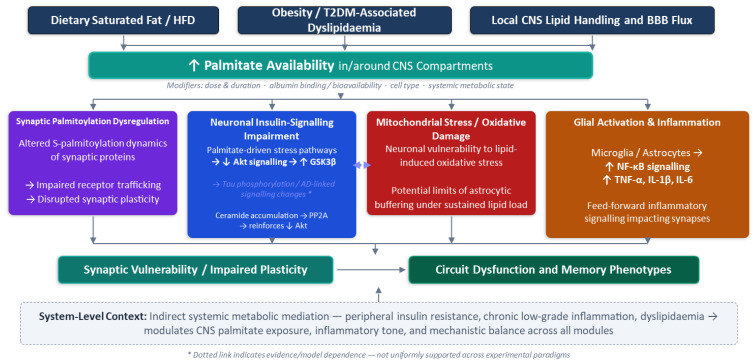
A model for palmitate-mediated neurotoxic pathways linking dietary saturated fat to CNS dysfunction. Three upstream factors (high-fat diet, obesity/T2DM-associated dyslipidaemia, and altered CNS lipid handling at the blood–brain barrier) converge to elevate palmitate availability within CNS. Increased palmitate drives four parallel mechanisms: (1) dysregulated S-palmitoylation of synaptic proteins, impairing receptor trafficking and plasticity; (2) neuronal insulin-signalling impairment via Akt/GSK3β, with downstream tau hyperphosphorylation; (3) mitochondrial stress and oxidative damage exceeding astrocytic buffering capacity; and (4) microglial/astrocytic activation amplifying NF-κB-driven pro-inflammatory cytokine release (TNF-α, IL-1β, and IL-6). All mechanisms converge on synaptic vulnerability and impaired plasticity, ultimately manifesting as circuit dysfunction and memory phenotypes in vivo. Dashed box: the systemic metabolic context (peripheral insulin resistance, chronic inflammation, and dyslipidaemia) modulates CNS exposure and inflammation across all modules.

Table 1 summarises selected palmitate-focused studies discussed in this section, including model system (dietary vs. in vitro exposure) and proposed mechanistic pathway, to aid comparison across heterogeneous paradigms. A graphical summary of this scheme is presented in Figure 1.

#### 2.2.3. Hippocampal Neurogenesis

Neurogenesis is the process whereby new neurones are generated from neural stem cells [90]. Adult hippocampal neurogenesis (AHN) was first reported in the 1960s in animals and has since been confirmed by immunohistochemistry in humans [91]. This process occurs in two neurogenic niches, namely, the subventricular zone (SVZ) of the lateral ventricles and the dentate gyrus (DG) of the hippocampus, and is thought to have an important role in learning and memory (for reviews, see [90]). Critically, AHN has been shown to decrease in AD [90], and enhancing this process can ameliorate memory deficits in AD mice [92,93,94]. Dysfunctional neurogenesis has also been reported in obesity and non-obesity T2DM mouse models [95], suggesting that metabolic and dietary factors can affect this process.

##### Diet as a Regulatory Factor in AHN

AHN can be influenced by diet, indicating that the defective neurogenesis observed in a T2DM mouse model could be at least partly due to this [96]. One study found that HFD decreased the number of new neurones in the dentate gyrus of female but not male mice [97]. Intriguingly, another study demonstrated that maternal HFD caused reduced neuronal stem/progenitor cell proliferation in offspring for two generations [98], implying epigenetic changes with profound impacts on future generations. Another study demonstrated that 12 weeks of a high-fructose diet caused the inhibition of synaptic plasticity, memory and hippocampal neurogenesis in rats [99]. However, another study showed that whilst a hypercalorific diet resulted in impaired synaptic plasticity and memory formation, it had no effect on hippocampal neurogenesis in aged rats [100]. These discrepancies likely occur from age at exposure, diet composition, strain differences and methods used to assess adult neurogenesis.

In line with previous subsections, palmitate specifically has been implicated in this reduction in AHN. Male C57BL/6 mice fed a palmitate-rich diet for 2 weeks showed reduced neural progenitor cell proliferation without affecting differentiation or glial activation, suggesting that palmitate disrupts the proliferation stage [101], and other studies reported similar effects [102]. Mechanistically, both these studies suggested that the reduction in AHN was due to direct lipotoxicity causing increased ROS. Interestingly, a more recent study demonstrated epigenetic reprogramming induced by palmitate exposure [103], which might explain the multigenerational observations of Natale et al. [98]. Conversely, another study found that high levels (250 µM) of palmitate resulted in increased differentiation of human embryonic stem cells into cortical neurones [104]. Thus, there is some uncertainty around the effect of palmitate specifically on AHN—however, the consensus seems to be that an HFD reduces AHN. This correlates well with the known effects of HFD on learning and memory, and provides another mechanism for the link between metabolic health and neurodegeneration.

A recent study from our group, using the neuroblastoma cell line SH-SY5Y as a model for neuronal differentiation/maturation (Targett et al. 2024), demonstrated that chronic, low-level exposure to palmitate (20 µM, the same level as observed by Melo et al. 2020 [61]) dysregulated this process [89]. Importantly, the deleterious effects of palmitate were only seen if the cells were exposed throughout the differentiation process, implying that differentiating cells are uniquely vulnerable to fatty acid-induced defects. Thus, it is possible that the apparent discrepancies in results may be due to differing exposure windows or unphysiological doses of fatty acids.

##### Reflections on Translational Considerations

Compared with the largely preclinical evidence for specific lipids and acute exposure paradigms, the most robust human-relevant evidence is the consistent association between metabolic disorders (notably, type-2 diabetes and metabolic syndrome) and later cognitive decline, alongside evidence that improving metabolic health is beneficial for multiple health outcomes that plausibly intersect with brain health. However, direct evidence that dietary interventions prevent or reverse neurodegenerative pathology in humans is still limited, and studies vary widely in their approaches and readouts.

Future clinical studies will likely be most informative when they combine cognitive assessments with metabolic and inflammatory biomarkers (e.g., insulin sensitivity measures, lipid profiles, and cytokines), and—where feasible—neuroimaging or fluid biomarkers that reflect neuroinflammation or synaptic integrity. Preclinical work can support these efforts by defining which mechanistic pathways are most consistent across models and which exposure paradigms are most physiologically anchored.

Table 2 provides selected examples across dietary paradigms (high-fructose, high-sucrose, and high-fat diets) to illustrate common synaptic and behavioural effects and to highlight where findings are sensitive to age, duration and metabolic state. For obvious reasons, such controlled studies are only possible in animal models, and therefore their translational potential is questionable.

## 3. Caloric Restriction

So far, we have only looked at the deleterious neuronal effects of caloric excess—but what about the opposite? The beneficial effects of caloric restriction (CR, typically a 20–40% reduction in calorie intake without malnutrition) on increasing lifespan, inflammation and oxidative stress in a variety of animal models have been extensively reported [105]. However, there is increasing evidence of positive effects on neuronal and synaptic function.

Multiple studies in rodents have demonstrated that both CR and intermittent fasting (IF) increase hippocampal neurogenesis [106,107], reduce neuroinflammation [108,109] and have a positive effect on synaptic plasticity and memory formation [107,109,110]. The mechanisms behind this are still under debate; however, one recurrent effect of both CR and IF is an increase in BDNF in the hippocampus and cerebral cortex. BDNF has multiple roles, most of which are mediated via the TrkB receptor. These have been extensively reviewed elsewhere [111], but in summary it has well-evidenced roles in neuronal survival, hippocampal neurogenesis and synaptic plasticity. It also reduces neuroinflammation via negative regulation of NF-k-B signalling in microglia [112]. Therefore, whilst other mechanisms clearly play a role, potentially BDNF is the major mediator of positive neurological effects of CR/IF.

There are several proposed mechanisms by which CR or IF can result in increased expression of BDNF. Firstly, the mild metabolic stress induced by these diets increases cAMP levels in neurones via glucagon/orexin signalling. This increases the phosphorylation of a key neuronal transcription factor, CREB, which in turn leads to increased expression of BDNF [106]. Secondly, the activation of AMP-sensitive protein kinase (AMPK) leads to phosphorylation and activation of the transcriptional coactivator, PGC1α, and is associated with increased hippocampal BDNF [113]. Thirdly, reduced energy intake leads to a shift towards ketogenesis, increasing levels of β-hydroxybutyrate (BHB), which induces BDNF expression via increased CREB phosphorylation and histone acetylation [114]. Interestingly, it has also been shown that BHB can mitigate against some of the effects of high-fat diet [115], possibly also by this mechanism.

## 4. Future Directions

Whilst it is clear from this review that there are multiple lines of evidence linking diet to neurological function, in many cases the exact mechanisms are uncertain. It is also true that, whilst the experimental evidence of a link is clear, the relevance of this to human health and disease is less so. Notable gaps in our knowledge include the levels of free fatty acids in the human brain. What concentration of fatty acids do neurones ‘see’, and how does this relate to their function? Certainly, there will be crosstalk between neurones, astrocytes and microglia in the handling of fatty acids, but these processes are still poorly understood. A greater understanding of the complex relationships between the different cells of the brain, in terms of metabolism and response to metabolic challenges, will greatly aid our understanding of the link between diet and neuronal function in health and disease.

While much of the mechanistic literature discussed here is weighted toward Alzheimer’s disease models, altered lipid handling, insulin signalling, mitochondrial stress and neuroinflammation are also implicated across other neurodegenerative conditions, including Parkinson’s disease and ALS. Importantly, dietary fat manipulation is not uniformly detrimental across contexts; some studies report protective effects of high-fat or ketogenic-like paradigms in specific disease models and outcomes. A key priority is therefore to define which disease stages, mechanisms and patient subgroups might plausibly benefit from metabolic interventions, rather than assuming a single-direction effect of “high fat” across disorders.

## 5. Conclusions

The effect of altered, ‘unhealthy’ diets on the brain is obviously complex. This is hardly surprising; the brain is an extremely complex organ with many different cell types, which have multiple metabolic pathways and all of which communicate with and depend on each other. Therefore, fuel supply to the brain is likely to have profound effects on the functioning of these cells, especially if that fuel supply falls outside normal parameters, e.g., a high-fat or high-sugar diet. Whilst there is abundant evidence, both from animal models and human studies, that these diets have a deleterious effect on the brain, and indeed may increase the risk of certain neurodegenerative diseases, the exact mechanisms are somewhat unclear. Certainly, there is a role for specific molecular players; e.g., neuroinflammatory cascades, insulin resistance, oxidative stress, and altered post-translational modifications. However, linking these effects to specific dietary components, particularly in humans, is always a great challenge. This is further complicated by the clear influence of biological sex in several of the studies detailed here.

Across the diverse evidence base reviewed here, two recurring mechanistic themes—insulin resistance-linked signalling changes and neuroinflammatory amplification—appear repeatedly across models and may represent common interfaces through which dietary and metabolic perturbations can influence synaptic vulnerability. However, the apparent ‘convergence’ of these themes reflects synthesis across heterogeneous evidence tiers (human observational, animal in vivo and cellular/ex vivo models), rather than a single demonstrated causal pathway in humans. These processes plausibly interact with oxidative stress, altered lipid trafficking/palmitoylation, ceramide accumulation, and cell-type-specific metabolic handling (astrocytes, neurons and microglia), with outcomes shaped by exposure duration, diet composition, age and sex. At the same time, key uncertainties remain regarding physiological CNS exposure levels and the extent to which commonly used experimental paradigms model human-relevant conditions. Addressing these gaps—and aligning preclinical endpoints with realistic clinical biomarkers—will be essential for translating mechanistic insight into actionable strategies for reducing neurodegenerative risk.

## Figures and Tables

**Table 1:** Selected examples of palmitate-associated effects on neuronal/glial endpoints, highlighting model system and inferred mechanism (predominantly animal/cellular evidence).

Study	Effects of Palmitate	Evidence Tier	Conditions/Delivery	Mechanism Proposed
Melo et al. 2020 [61]	Correlation with cognitive decline + memory impairments	Human in vivo (observational) Animal in vivo (mouse)	Observational (human) Intracerebrovascular infusion of palmitate (mouse)	TNFα activation, hippocampal inflammation
Rojas et al. 2025 [66]	↓ GluA1 surface expression, ↓ synaptic plasticity	Animal in vivo (mouse)	Dietary (7% calories from palmitate)	Postsynaptic, potentially changes in GluA1 palmitoylation
Spinelli et al. 2017 [32]	↓ hippocampal synaptic plasticity ↓ memory	Animal in vivo (mouse) Animal ex vivo (isolated cells)	HFD (60% saturated fat) 200 µM (isolated cells)	↑ palmitoylation of GluA1, insulin resistance
Amine et al. 2021 [68]	Insulin resistance + ↑ neuroinflammation	In vitro (undifferentiated SH-SY5Y cell line)	200 µM direct treatment (4 h)	↑ TLR4 expression, ↑TNFα
Sánchez-Alegría et al. 2021 [69]	Insulin resistance	In vitro (undifferentiated MSN cell line)	200 µM, direct exposure	↑ calcium entry via stimulation of ATP production and K_ATP_ channel closure
Yang et al. 2022 [82]	↑ inflammatory markers	In vitro (BV2 cell line)	20–160 µM	Increased TLR4/NF-kB signalling/p65 signalling
Chmielarz et al. 2023 [83]	↑ inflammatory markers (IL-6 and MCP-1)	In vitro (HMC3 cell line)	200 µM	Potentially JAK/STAT signalling, lipid peroxidation and oxidative stress
García-Cruz et al. 2024 [78]	↑ tau phosphorylation	In vitro (differentiated MSN cell line)	200 µM, 1–24 h	↑ GSK3β and mTOR activity
Targett et al. 2026 [89]	Inhibition of neuronal differentiation	In vitro (differentiated SH-SY5Y cell line and hIPSC-derived forebrain neurones)	20 µM, 10-day treatment	Insulin resistance, dysregulation of CREB, GSK3β and CDK5 activity
Zhou et al. 2023 [84]	↑ palmitate ↑ inflammatory signalling (multiple cytokines) in microglia	In silico meta-analysis	N/A	Multiple pathways including TNFα, NF-kB, and TLR4

**Table 2:** Selected diet-based studies and key findings derived from animal models.

Dietary Factors	Study	Observations
High-fructose diet	Cisternas et al. 2015 [24]	Reduction in LTP/LTD, reduced performance in MWM, reduction in neurogenesis, and insulin resistance.
	Kovačević et al. 2024 [31]	↑ CaMKII phosphorylation, altered synaptic protein expression, and increased stress behaviour.
High-sucrose diet	Coirini et al. 2022 [37]	Memory defects, ↓ hippocampal BDNF, and metabolic dysfunction. Specific to juvenile exposure.
	Hernández-Ramírez et al. 2022 [36]	Several different memory defects. Defective hippocampal LTP.
	Kruse et al. 2019 [34]	Increased anxiety and reduced exploring behaviour (when rats are exposed as juveniles).
	Davis et al. 2020 [35]	Circadian changes, impaired spatial working memory, and reduced hippocampal GluN2B expression.
High-fat diet	McLean et al. 2018 [44]	Episodic memory impairment by short term HFD (reversible).
	Spencer et al. 2017 [45]	Hippocampal and amygdalar memory deficits. Neuroinflammation.
	Chiazza et al. 2021 [46]	Reduced dendritic complexity in immature hippocampal neurones. Reduction in BDNF levels. Short-term HFD (reversible).
	Wu et al. 2024 [47]	Depressive-like behaviour and reduced Wnt5a signalling, reversible by exercise.
	Yang et al. 2025 [52], Bracko et al. 2020 [51]	Exacerbation of AD pathology in model mice (increased Aβ, neuroinflammation, amyloid plaques and memory deficits).
	Robison et al. 2020 [97]	Decreased AHN in female but not male mice.
	Natale et al. 2022 [98]	Multigenerational effect of maternal HFD on neurogenesis in offspring.

## Data Availability

Not applicable.
